# Combined Metabolome and Transcriptome Analyses Unveil the Molecular Mechanisms of Fruit Acidity Variation in Litchi (*Litchi chinensis* Sonn.)

**DOI:** 10.3390/ijms24031871

**Published:** 2023-01-18

**Authors:** Yonghua Jiang, Yingwei Qi, Xilong Chen, Qian Yan, Jiezhen Chen, Hailun Liu, Fachao Shi, Yingjie Wen, Changhe Cai, Liangxi Ou

**Affiliations:** 1 Institute of Fruit Tree Research, Guangdong Academy of Agricultural Sciences/Key Laboratory of South Subtropical Fruit Biology and Genetic Resource Utilization, Ministry of Agriculture and Rural Affairs/Guangdong Key Laboratory of Tropical and Subtropical Fruit Tree Research, Guangzhou 510640, China; 2 Sericultural & Agri-Food Research Institute Guangdong Academy of Agricultural Sciences, Key Laboratory of Functional Foods, Ministry of Agriculture and Rural Affairs, Guangdong Key Laboratory of Agricultural Products Processing, Guangzhou 510610, China; 3 Quantitative Genetics and Evolution Laboratory, Paris-Saclay University/INRAE/CNRS/AgroParisTech/GQE–Le Moulon, 91190 Gif-sur-Yvette, France

**Keywords:** litchi, fruit acidity, fumarate, GABA, metabolomics, transcriptome sequencing, malate metabolism

## Abstract

Fruit acidity determines the organoleptic quality and nutritive value of most fruits. In litchi, although the organic acid composition of pulps is known, the molecular mechanisms and genes underlying variation in fruit acidity remain elusive. Herein, developing pulps of two contrasting litchi varieties, Huaizhi (HZ, low-acidity) and Boye_No.8 (B8, high-acidity), were subjected to metabolomics and transcriptomics, and the dynamic metabolome and transcriptional changes were determined. Measurements revealed that the dominant acidity-related organic acid in litchi pulps is malate, followed in low levels by citrate and tartrate. Variation in litchi pulps’ acidity is mainly associated with significant differences in malate and citrate metabolisms during fruit development. Malic acid content decreased by 91.43% and 72.28% during fruit ripening in HZ and B8, respectively. The content of citric acid increased significantly in B8, while in HZ it was reduced considerably. Differentially accumulated metabolites and differentially expressed genes analyses unveiled fumarate, succinate, 2-oxoglutarate, GABA (γ-aminobutyric acid), phosphoenolpyruvate, and citrate metabolisms as the key driving pathways of litchi fruits’ acidity variation. The drastic malate and citrate degradation in HZ was linked to higher induction of fumarate and GABA biosynthesis, respectively. Thirty candidate genes, including three key genes (*LITCHI026501.m2*, fumarase; *LITCHI020148.m5*, glutamate decarboxylase; and *LITCHI003343.m3*, glutamate dehydrogenase), were identified for functional studies toward genetic modulation of litchi fruit acidity. Our findings provide insights into the molecular basis of acidity variation in litchi and provide valuable resources for fruit quality improvement.

## 1. Introduction

Fleshy fruits are the most in demand fruits worldwide due to their organoleptic qualities, therapeutical attributes, and nutritional values [1]. Fruits’ taste is generally divided into sour and sweet and is mainly related to the composition and concentration of organic acids and soluble sugars. Of them, acidity is the trait that affects fruit quality and nutritive values the most [2,3]. A higher quantity of acids often reduces fruit quality, while a moderate acids content can enhance fruit palatability [4]. Fruit acidity is primarily caused by the accumulation of organic acids, mainly malate, citrate, tartrate, and quinate, in the fleshy parts of fruits [5,6]. The content of organic acids in fruit undergoes great variations during development and ripening depending on genetic and environmental factors [2,5,6,7]. Thus, understanding the molecular mechanisms involved in organic acid metabolism in fruits is of great interest for improving fruits’ quality and commercial values.

As with many developmental processes, fruit ripening is well coordinated and requires changes in the expression of thousands of genes to modulate various physiological and biochemical signal cascades, including organic acids metabolism [8]. The accumulation of organic acids in fruit cells is a complex phenomenon that involves diverse interlinked metabolic pathways and transport mechanisms across many compartments (cytosol, mitochondria, vacuole, peroxisome, etc.) and is under the control of various factors [5,9]. In many fruit plant species, aluminum-activated malate transporter (ALMT), NAD-malate dehydrogenase (NAD-MDH), pyruvate kinase (PK), pyruvate dehydrogenase kinase (PDK), glutamate decarboxylase (GAD), alcohol dehydrogenase (ADH), and other acid-metabolism-related enzymes have been identified to affect fruit acidity through the synthesis or degradation of organic acids [1,2,3,8,10]. In peach, low acidity is associated with higher expression of a GAD gene and citrate degradation through GABA (γ-aminobutyric acid) shunt [1,3]. GABA is a signaling molecule in plants that can affect tricarboxylic acid metabolism through negative regulation of anion flux via ALMT [11,12]. Moreover, studies in *Arabidopsis* have revealed that fumarase (FUM, fumarate hydratase) activity is regulated in a multilevel manner, and FUM plays an essential role in carbon allocation via organic acids metabolism in plants [13,14]. Therefore, a higher induction of fumarate biosynthesis and accumulation may also influence fruits’ organoleptic quality. Moreover, dissecting acidity-related mechanisms in diverse fruit plant species will deepen the global understanding of fruits’ taste variation and help in designing an overall scheme for targeted modulation of fruit quality.

Litchi (*Litchi chinensis* Sonn.) is an evergreen subtropical fruit tree. It is a member of the Sapindaceae family and is native to southern China (Guangdong, Hainan, Fujian, etc.) and northern Vietnam [15,16]. Litchi fruits are popular and attractive with a red color, palatable flavor, and an increasing commercial value [17,18]. Sugars are the predominant nutrients in litchi pulp, and sugar-associated molecular mechanisms have been largely investigated [19,20]. Studies have shown that the main organic acids in litchi pulp are malic, tartaric, citric, and ascorbic acids, with malic acid accounting for ~80% of the total organic acids [19,21,22]. In a recent study, Haizhi et al. investigated the dynamic changes in the content of major organic acids in Feizixiao, one of the common litchi cultivars in China, and have provided an overview of the expression patterns of organic acid-related genes during fruit ripening in this variety [22]. However, no insight was provided regarding the molecular mechanisms that underly variation in litchi fruit acidity, and key candidate genes still need to be determined. Hence, there is a need to perform an integrated analysis of the dynamic metabolome and transcriptome changes during litchi fruit development and to identify key differentially regulated metabolites, genes, and pathways. This will provide important resources for molecular control of litchi fruits’ acidity. These approaches have been broadly used to decipher organic acid metabolism in diverse species [1,2,5,8]. 

In the present study, we analyzed the dynamic changes in major organic acids in litchi pulp of two contrasting varieties, Huaizhi (HZ, low-acidity) and Boye_No.8 (B8, high-acidity), at five fruit developmental stages. Moreover, we combined metabolomics and transcriptomics analyses on the same developing fruit pulps and uncovered key metabolic pathways that drive acidity variation in litchi.

## 2. Results

### 2.1. Dynamic Changes in the Content of Major Organic Acids in Litchi Pulp during Fruit Development

**Figure 1:**
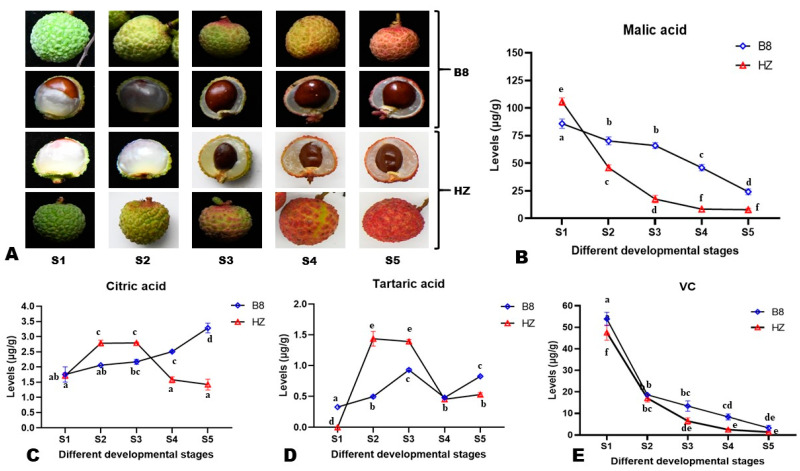
Morphological observation of litchi fruits and pulps from HZ and B8 and different developmental stages (**A**). Overview of the dynamic changes in the content of malic acid (**B**), citric acid (**C**), tartaric acid (**D**), and VC (**E**) in HZ and B8 during fruit ripening. S1, S2, S3, S4, and S5 represent different developmental stages. Each value is expressed as mean ± standard error (n = 3). Different letters display significant (*p* < 0.05) differences (one-way ANOVA) for each organic acid at the different fruit developmental stages in the two varieties.

To examine changes in the content of major organic acids in the pulp of litchi during fruit development, pulp samples from five different developmental stages (S1–S5, Figure 1A and Figure S1) of two litchi varieties, Huaizhi (HZ, low-acidity) and Boye_No.8 (B8, high-acidity), were subjected to HPLC analysis. In total, four organic acids, including malic acid, citric acid, ascorbic acid (VC, vitamin C), and tartaric acid, were detected and quantified (Figure 1B–E). The results showed that the difference in HZ and B8 pulps’ acidity is mainly related to differences in malate and citrate metabolisms during fruit development. At S1, the malic acid content in HZ was significantly (*p* < 0.05) higher than in B8 (Figure 1B). However, from S2 to S5, the malic acid content of HZ pulps drastically decreased to a low level compared to B8 (Figure 1B). For instance, the content of malic acid in HZ was significantly reduced by 91.43% (*p* < 0.0001, from 106.2 to 9.1 µg/g), while in B8, it decreased from 85.5 to 23.7 µg/g (72.28%). The citric acid content of B8 pulps increased significantly along with fruit development in contrast to HZ (Figure 1C). In HZ pulps, the content of citric acid was significantly increased from S1 to S2, almost stable from S2 to S3, then considerably reduced from S3 to S5 (Figure 1C). There was no statistical difference between the citric acid content in B8S1 and HZS5 (Figure 1C), indicating that citric acid degradation is highly induced in HZ at late stages of fruit development. The tartaric acid content of HZ pulps significantly increased from S1 to S2, then it was reduced considerably from S2 to S4 (Figure 1D). At S5, the contents of citric acid and tartaric acid in B8 pulps were 3.2 and 0.8 µg/g, respectively, compared to 1.4 and 0.5 µg/g, respectively, in HZ (Figure 1C,D). The VC content exhibited similar trends in the pulps of the two varieties along with fruit development, with a significant decrease from S1 to S2 and a slight reduction from S2 to S5 (Figure 1E).

### 2.2. Diversity and Variation of Metabolites in Litchi Pulp during Fruit Development

**Figure 2:**
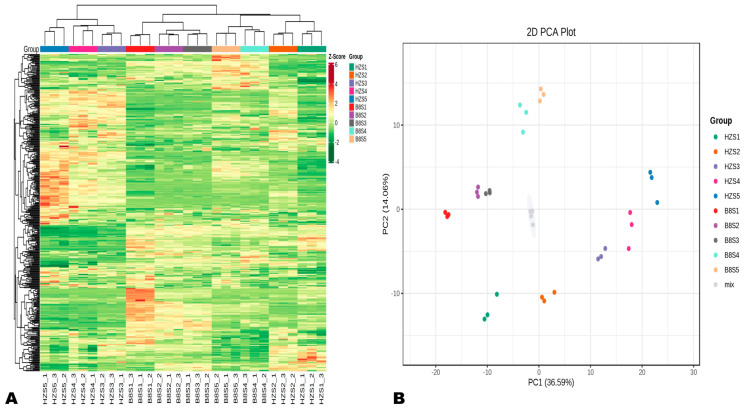
Overview of dynamic changes and variation in HZ and B8 pulps’ metabolome during fruit ripening. (**A**) Hierarchical cluster analysis (HCA) results exposing variation between and within the groups. (**B**) Principal component analysis (PCA) results showing metabolite profile differences between and within the groups.

To explore dynamic metabolism changes during HZ and B8 fruit development, we performed UHPLC-MS/MS-based widely targeted metabolomics analysis on their respective pulp samples from the five developmental stages. In total, 401 metabolites in litchi pulp were detected and structurally annotated (Table S1). Principal component analysis (PCA) and hierarchical cluster analysis (HCA) indicated that the metabolite profiles of HZ and B8 pulps were very different and varied along with the fruit development (Figure 2A,B). Samples in the same group clustered together, indicating the repeatability of the experiment. In the PCA plot, the four duplicate QC samples were gathered together close to the plot center, confirming that the experiment is repeatable and reliable (Figure 2B). Supportively, OPLS-DA analysis results indicated the strong goodness of fit (R2X > 0.62, R2Y> 0.99) and the high predictability (Q2 > 0.96) of the models (Figure S2).

**Figure 3:**
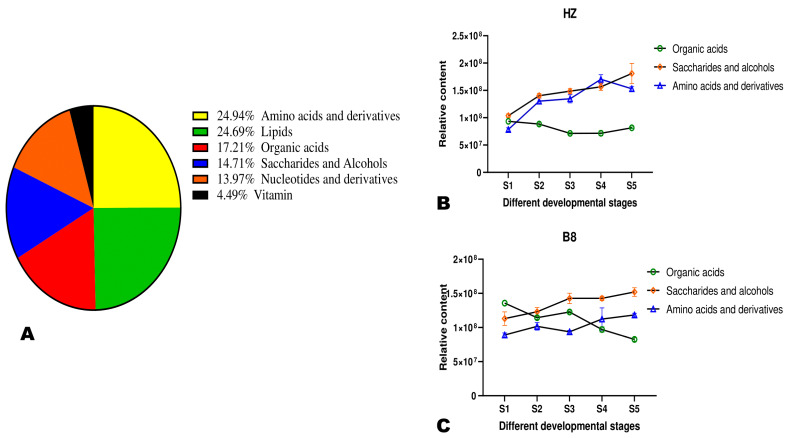
(**A**) Classification of the 401 identified metabolites in pulps of litchi fruit. (**B**,**C**) Dynamic changes in the total relative content of organic acids, saccharides and alcohols, and amino acids and derivatives in HZ and B8 pulps during fruit ripening, respectively.

Metabolites’ classification showed that the primary metabolites in litchi pulp were amino acids and derivatives (24.94%), followed by lipids (24.69%), organic acids (17.21%), saccharides and alcohols (14.71%), nucleotides and derivatives (13.97%), and vitamins (4.49%) (Figure 3A). Organic acid metabolism in plants is interconnected mostly with sugars and amino acid metabolisms [23,24]. We investigated changes in these three classes of metabolite accumulation in HZ and B8 pulps during fruit development (Figure 3B,C). In the two varieties, the accumulation of amino acids and saccharides and alcohols exhibited an increased tendency along with fruit development, albeit their respective relative contents were slightly higher in HZ than in B8. The relative total organic acids content in B8 decreased with fruit maturity, while no major fluctuation was observed in HZ (Figure 3B,C), suggesting that the above-recorded decrease in malic and citric acids and HZ might be due to their conversion into other organic acids. Supportively, K-means analysis revealed that the content of fumaric acid and γ-aminobutyric acid (GABA) increased along with seed development in HZ (Figure S3 and Table S2). These two organic acids are directly linked to malate metabolism and have less of an acidity effect on fruit taste [5,23,24]. 

### 2.3. Differentially Accumulated Metabolites (DAMs) in HZ and B8 Pulps

**Figure 4:**
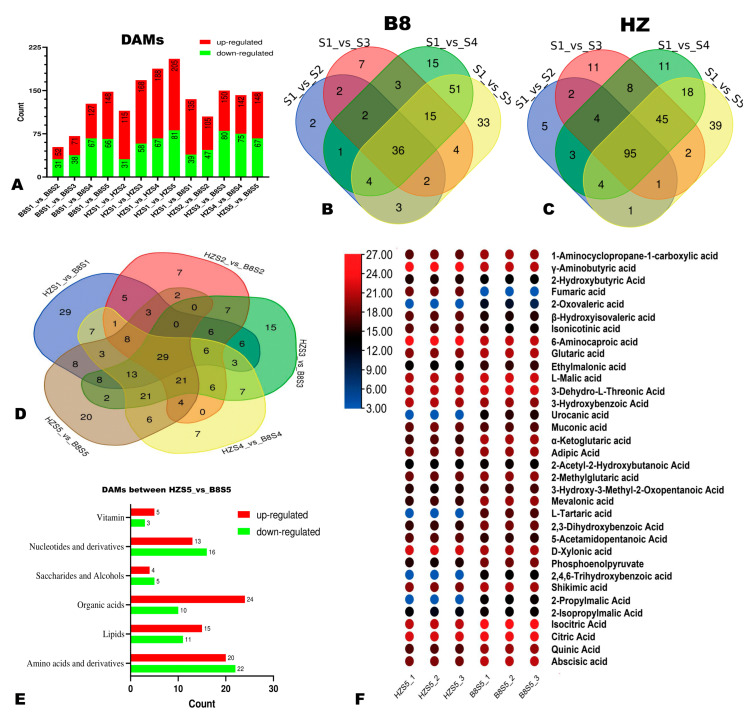
Differentially accumulated metabolites (DAMs) between HZ and B8 during fruit ripening. (**A**) Summary of the number of DAMs during fruit development in B8 and HZ and between the two varieties. (**B**,**C**) Venn diagram showing the number of DAMs in B8 and HZ along with fruit development, respectively. (**D**) Venn diagram of the DAMs between HZ and B8 at the five fruit developmental stages. (**E**) Classification of the 148 DAMs between HZ and B8 at the red ripening stage (S5). (**F**) Heatmap of relative content of differentially accumulated organic acids between HZ and B8 at S5.

We applied the thresholds of *p*-value < 0.05 and VIP ≥ 1 to identify the significant DAMs in HZ and B8 pulps along with fruit development. We found that the number of DAMs in the pulps of the two varieties increased along with the fruit development (Figure 4A–C). Compared to B8, there were more DAMs, especially upregulated metabolites in HZ (Figure 4A), indicating that many changes had occurred in HZ fruit metabolism along with maturation in comparison to B8. The DAMs between HZS1 and HZS5 and between B8S1 and B8S5 are presented in Tables S3 and S4, respectively. It was noteworthy that malic acid and isocitrate acid were downregulated, while fumaric acid was upregulated in HZ (Table S3). In contrast, these organic acids were not figured among the DAMs in B8 (Table S4). Regarding metabolome differences between the two varieties, there were 135 (96 upregulated), 105 (58 upregulated), 150 (70 upregulated), 142 (67 upregulated), and 148 (81 upregulated) significantly differential metabolites between HZ and B8 at S1, S2, S3, S4, and S5, respectively (Figure 4A and Figure S4). Of them, 29 metabolites, including seven organic acids, were significantly differentially accumulated in the pulps of the two varieties along with the fruit development (Figure 4D). The seven organic acids are highlighted in Table S5 and include GABA, phosphoenolpyruvate (PEP), glutaric acid, etc. Fumaric acid and 2-oxoglutaric acid were down- and upregulated, respectively, in B8 pulp from S2 to S5 in contrast to HZ. Table S5 presents the list of DAMs between HZ and B8 at S5. In total, 34 organic acids, including 24 upregulated and 10 downregulated metabolites, were identified in the pairwise comparison between HZ and B8 at S5 (Figure 4E). We examined the relative content of the 34 differentially accumulated organic acids, and the results revealed that fumaric acid was significantly downregulated in B8 while it was upregulated in HZ (Figure 4F).

### 2.4. Transcriptome Profiles of HZ and B8 Pulps during Fruit Development

In order to identify the key molecular mechanisms related to variation in litchi fruit acidity, we carried out comparative transcriptome analyses of their respective pulp samples at the five developmental stages. In general, we generated 43.3 to 45.57 Mb of raw data from each of the thirty pulp samples (Table S6). After eliminating splice sequences, low-quality reads, and uncertain reads, the total clean reads ranged from 41.82 to 43.61 Mb. The Q20 and Q30 values varied from 95.19 to 96.9% and 89.3 to 92.22%, respectively, indicating that the RNA-seq data were of high quality. The clean reads were further mapped into the litchi genome, and the achieved mapping rates ranged from 72.33 to 81.09% (Table S6). We performed PCA analysis to differentiate between samples of different groups (Figure S5). The results indicated that the transcriptomes of HZ and B8 were very different and changed with fruit development. Samples in the same group were gathered together in the PCA plot, confirming the repeatability of the experiment.

### 2.5. Differentially Expressed Genes (DEGs) between HZ and B8

**Figure 5:**
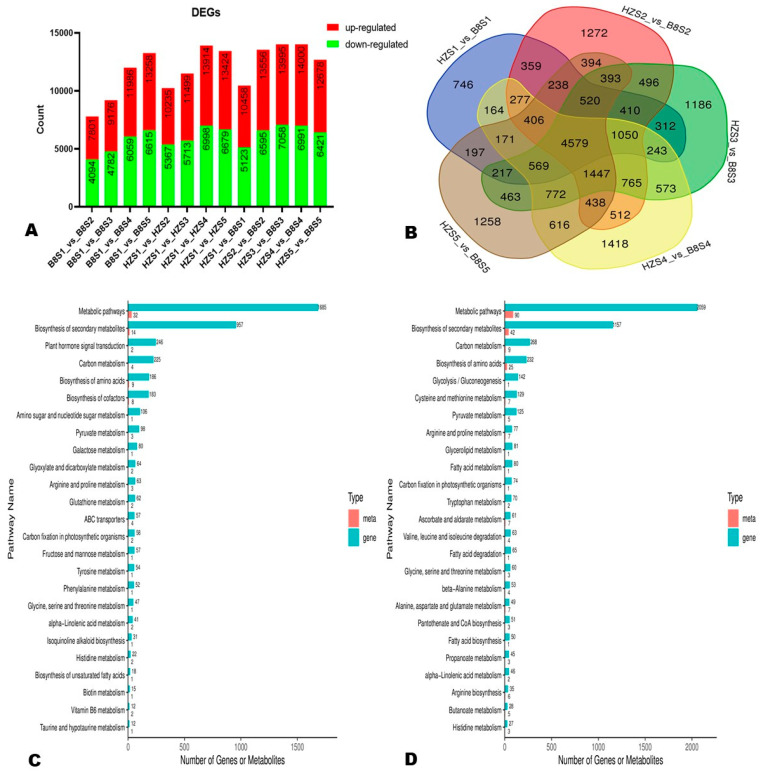
Differentially expressed genes (DEGs) between HZ and B8 during fruit ripening. (**A**) Summary of the number of DEGs during fruit development in B8 and HZ and between the two varieties. (**B**) Venn diagram of DEGs between HZ and B8 at the five fruit developmental stages. (**C**,**D**) KEGG annotation results of the DEGs between B8S1 and B8S3; and between HZS1 and HZS3, respectively. Meta and gene indicate metabolites and DEGs, respectively.

To examine the transcriptional differences between HZ and B8 and in samples from different developmental periods, we screened out DEGs via thresholds *p*-value < 0.05 and |log2FoldChange| > 1. In the two varieties, the number of DEGs increased along with fruit development (Figure 5A). At S1, S2, S3, S4, and S5, we identified 10,458 (5335 upregulated), 13,556 (6961 upregulated), 13,995 (6937 upregulated), 14,000 (7009 upregulated), and 12,678 (6257 upregulated) DEGs, respectively, between the two varieties (Figure 5A). Of them, 4579 DEGs were identified at all of the developmental stages (Figure 5B).

**Figure 6:**
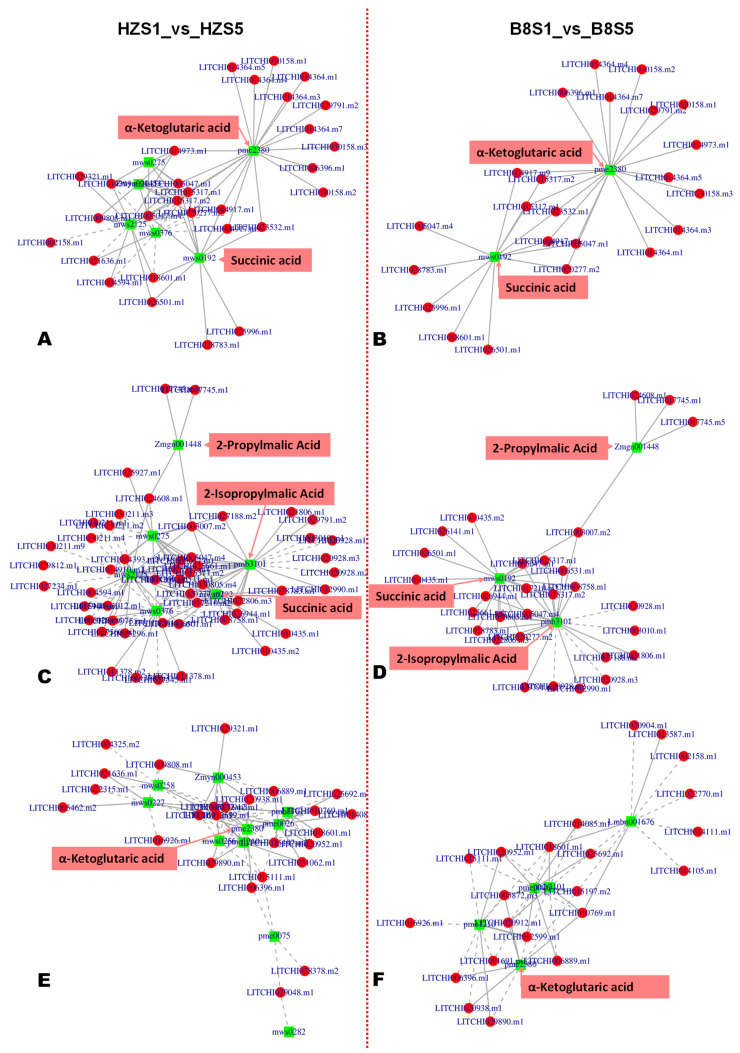
Metabolite–gene networks analysis results of key enriched KEGG pathways of organic acid metabolism. (**A**,**B**) TCA cycle between HZS1 and HZS5; and between B8S1 and B8S5, respectively. (**C**,**D**) Pyruvate metabolism between HZS1 and HZS5; and between B8S1 and B8S5, respectively. (**E**,**F**) 2-oxoglutarate metabolism between HZS1 and HZS5; and between B8S1 and B8S5, respectively.

Based on the malic acid and citric acid accumulation trends in the two varieties during fruit development, we selected the DEGs and DAMs between S1 and S3 and between S1 and S5 for functional annotation and to gain insight into the molecular mechanisms that underly litchi fruits’ acidity variation. We mainly focused on pathways related to malate and citrate metabolisms in plants [5]. The results revealed that 186, 98, 80, 64, 62, and 57 DEGs between B8S1 and B8S3 were involved in the biosynthesis of amino acids, pyruvate metabolism, galactose metabolism, glyoxylate and dicarboxylate metabolism, glutathione metabolism, and fructose and mannose metabolism, respectively (Figure 5C, Table S7). Meanwhile, the DEGs between HZS1 and HZS3 were involved in the biosynthesis of amino acids (232 DEGs), glycolysis/gluconeogenesis (142 DEGs), pyruvate metabolism (125 DEGs), ascorbate and aldarate metabolism (61 DEGs), and the metabolism of diverse amino acids (>500 DEGs) (Figure 5D, Table S8). Similar differences were observed at S5 (Figure S6). By examining these results, we found that the main molecular mechanisms that affect litchi fruits’ acidity were fumarate (mws0376), GABA (pme3011), succinate (mws0192), 2-oxoglutarate (α-ketoglutarate, pme2380), PEP (mws2125), and isocitrate (Zmyn000453) metabolisms. The metabolites-–genes correlation networks of key pathways, including TCA, pyruvate metabolism, and 2-oxocarboxylic acid metabolism in the two varieties at S3, are shown in Figure 6.

### 2.6. Candidate Genes for Controlling Litchi Fruit Acidity

**Figure 7:**
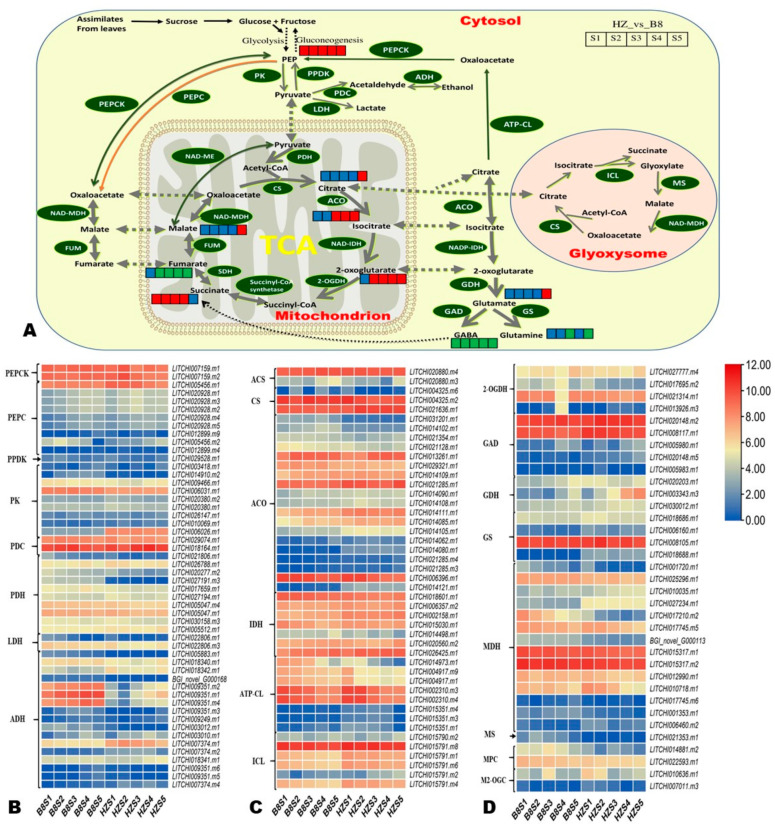
(**A**) Overview of the probable regulation of some key organic acids mapped to metabolic pathways in pairwise comparisons between HZ and B8 during fruit development. The red color small rectangle indicates that the metabolite content is significantly upregulated; the small green rectangle indicates that the metabolite content is significantly downregulated; the small blue rectangle indicates no significant difference in that metabolite’s content. (**B**–**D**) Heatmap of log2FC values of the expression of key differentially expressed organic acid metabolism genes in HZ and B8 during fruit development. PEP, phosphoenolpyruvate; PEPC, PEP carboxylase; PEPCK, PEP carboxykinase; PPDK, pyruvate orthophosphate dikinase; PK, pyruvate kinase; PDC, pyruvate decarboxylase; PDH, pyruvate dehydrogenase; LDH, lactate dehydrogenase; ADH, alcohol dehydrogenase, ACA, acetyl-CoA synthase; CS, citrate synthase; ACO, aconitase, IDH, isocitrate dehydrogenase; ATP-CL, ATP-dependent citrate lyase; ICL, isocitrate lyase; 2-OGDH, 2-oxoglutarate dehydrogenase; GAD, glutamate decarboxylase; GDH, glutarate dehydrogenase; GS, glutamine synthetase; MDH, malate dehydrogenase; MS, malic synthase; MPC, mitochondrial pyruvate carrier 2; M2-OGC, mitochondrial oxoglutarate transporter.

To facilitate the overview of key differentially regulated metabolic pathways affecting litchi fruit acidity, we constructed a map highlighting the majority of the DAMs between HZ and B8 based on the KEGG annotation and enrichment maps (Figure 7A). Furthermore, we manually screened out DEGs encoding key enzymes in these metabolic pathways based on previous studies [1,2,5,8,23,24]. In total, 171 DEGs were filtered out, and their expression patterns are shown in Figure 7B–D and Figure S7. Finally, based on the Log2FC values at the different developmental stages in the pairwise comparison between B8 and HZ, we selected 30 DEGs as potential candidate genes for functional studies toward genetic control of litchi fruits’ acidity (Table 1). It was noteworthy the gene *LITCHI026501.m2* that encodes fumarase (FUM, fumarate hydratase) was highly induced (|Log2FC| > 7.4) in HZ from S2 to S5 compared to in B8 (Table 1), indicating it might catalyze the conversion of malate into fumarate. The genes encoding glutamate decarboxylase (*LITCHI020148.m5*), glutamate dehydrogenase (*LITCHI003343.m3*), glutamine synthetase (*LITCHI018688.m1*), malate dehydrogenase (*LITCHI006460.m2*), 2-oxoglutarate dehydrogenase (*LITCHI013926.m3*), and aconitase (*LITCHI014080.m1* and *LITCHI014121.m1*), were upregulated in HZ compared to in B8 (Table 1). Compared to HZ*, LITCHI005883.m1, LITCHI009351.m2, LITCHI009351.m1*, and *LITCHI009351.m4* (alcohol dehydrogenase); *LITCHI014417.m1* (glutamate receptor, ionotropic, plant); *LITCHI021353.m1* (malate synthase); *LITCHI014881.m2* (mitochondrial pyruvate carrier 2); and *LITCHI027191.m3* (pyruvate dehydrogenase) were upregulated in B8 (Table 1).

### 2.7. Quantitative Real-Time-PCR (qRT-PCR) Validation

To verify the reliability of the RNA-seq data, we randomly selected fifteen of the thirty candidate genes for qRT-PCR analysis. As shown in Figure S8, the qRT-PCR results were consistent with the transcriptome data (Pearson correlation, r = 0.86), confirming the high confidence level of our findings.

## 3. Discussion

To enhance the economic value of many fruits, it is important to develop novel varieties with desired/improved organoleptic quality. Thus, there is a need to dissect and understand the genetic and molecular basis of fruit acidity, the trait that most affects fruit quality and nutritive values [2,3]. The present study unveiled key pathways and genes governing variation in litchi fruits’ acidity. Litchi is a functional food recognized worldwide for the management of diverse ailments [18]. Organic acids’ composition and concentration in litchi pulps vary depending on the genotypes and growing conditions, with the main components being malic acid and tartaric acid [21,22]. Accordingly, litchi fruits’ acidity varies considerably, influencing the fruits’ market and quality. Consistent with previous reports, we found that malic acid is the dominant organic acid in litchi pulps, followed by citric and tartaric acids. These acids exhibit different dynamic changes during fruit development in high-acid and low-acid varieties. The content of malic and citric acids was drastically reduced during fruit ripening in the low-acid variety, indicating that the low acidity of litchi fruits might be caused by considerable degradation of malate and citrate during fruit maturation. In contrast, in the high-acid variety, the content of citric acid increased during fruit ripening, and the malic acid content showed a moderate reduction. It is well known that fruit maturity of diverse varieties differentially affects metabolite accumulation and, in turn, fruit quality [25,26,27]. The association between low acidity and citric acid degradation during fruit ripening has been reported in peaches [1,3]. Taken together, these findings show that the regulation of organic acid metabolism differs regarding the genotype, and controlling malate and citrate metabolisms during fruit development may help produce litchi fruits with the desired taste and quality.

To provide insights into the mechanisms underlying fruits’ acidity variation in litchi, we performed metabolomics and transcriptomics analyses. These approaches have been broadly used to decipher organic acid metabolism in diverse species [1,2,5,8,28]. Metabolomics analyses revealed that the decrease in the content of organic acids during fruit ripening was accompanied by an increase in sugars and amino acid contents, suggesting that organic acids were metabolized in other metabolic pathways, such as neoglucogenesis and amino acid biosynthesis. These results are consistent with previous reports on other species [10,23,29]. However, it was noteworthy that the change in total organic acid during fruit development in the low-acid variety was very slight, although a drastic decrease in malate, citrate, and tartrate content was recorded. This result implies that these organic acids might be converted into other organic acids with very low acidity power in HZ. Supportively, DAMs analysis revealed that the major DAMs between HZ and B8 at the fruit maturity stage were fumarate, GABA, malate, citrate, isocitrate, 2-oxoglutarate, succinate, and PEP, with fumarate and GABA significantly upregulated in HZ. These results indicate that the low acidity of pulps of some litchi varieties is attributable to higher induction of fumarate and GABA metabolisms. Supportively, we found that 2-oxoglutarate was significantly upregulated in B8, inferring that a high transfer of 2-oxoglutaric acid from mitochondrion into the cytosol (where it is converted into glutamate) might have occurred during HZ fruit development. GABA is a non-proteinogenic amino-acid plant signaling molecule biosynthesized mainly through the decarboxylation of glutamate by GAD [30]. Its metabolism, labeled as the GABA shunt, bypasses two critical TCA cycle steps, resulting in citrate, isocitrate, and 2-oxoglutarate degradation [11,30,31]. In peaches, it has been demonstrated that the low acidity of fruits is associated with higher expression of a GAD gene and citrate degradation through GABA shunt [1,3]. Through the transcriptomics analyses, we found that two genes of the GABA pathway, *LITCHI020148.m5* and *LITCHI003343.m3,* encoding GAD and glutamate dehydrogenase, respectively, were significantly upregulated in HZ. Glutamate dehydrogenase catalyzes ammonium incorporation into 2-oxoglutarate to form glutamate [32]. Furthermore, GABA can control malic acid metabolism, especially its accumulation in the vacuole, via negative regulation of anion flux via ALMT [11,12,33]. ALMTs are a multigenic protein family involved in diverse physiological processes in plants [12,33]. They are activated by anions and are essential for malate transport and accumulation in vacuoles [2,11,28,34]. Together, these findings show that GABA metabolism positively regulates organic acid degradation during litchi fruit development, leading to low acidity of the pulps. Moreover, they infer that targeting GABA shunt genes might be an efficient approach to producing highly marketable litchi fruits with the desired organoleptic quality. Moreover, the identified DAMs could represent key markers for metabolomics-based selection of litchi varieties.

The study by Chia et al. showed that many C_3_ plants could accumulate higher amounts of fumaric acid which can be metabolized to produce energy and carbon skeletons for the biosynthesis of other compounds, such as soluble sugars and starch [35]. Other studies have shown that fumarate is a pH regulator, and targeting fumarate metabolism may offer great opportunities to enhance plant growth and production [36]. Herein, we found that the low-acid variety HZ accumulated a significantly higher quantity of fumarate in its pulps compared to B8, indicating that fumarate metabolism significantly contributes to litchi fruits’ acidity variation. In *Arabidopsis*, it was demonstrated that fumarate modulates the activity of the NADP-malic enzyme (NADP-ME) depending on the cytosolic pH [14]. Fumarate positively stimulates the activity of NADP-ME by binding to a specific allosteric site, resulting in the decarboxylation of malate into pyruvate [14]. Consistency, we found that the gene *LITCHI026501.m2*, which encodes FUM, was significantly induced (more than seven-folds) in HZ compared to B8. This gene may catalyze malate dehydration into fumarate, occasioning high accumulation of fumarate in low-acid litchi varieties. In *Arabidopsis*, it was demonstrated that the malate dehydratase (MD) activity of FUM is preferred over fumarate hydratase activity, as MD activity is positively regulated by multiple allosteric activators, including asparagine, glutamine, and oxaloacetate [13]. In addition, asparagine and glutamine were upregulated in HZ. These results indicate that *LITCHI026501.m2* is essential for massive fumarate accumulation in pulps of low-acid litchi varieties and that it is a modulator of carbon flow via organic acid metabolism in litchi. The multilevel regulatory pattern of FUM activities supports the pivotal role of this enzyme in monitoring carbon flow through the metabolism of organic acids in plants [13]. Functional characterization of *LITCHI026501.m2* is required to exploit its full potential in improving litchi fruit quality.

Moreover, we have identified other candidate genes that may also contribute to pulps’ acidity variation in litchi. Of them, *LITCHI004325.m6*, *LITCHI017210.m2*, *LITCHI014881.m2*, *LITCHI021353.m1*, *LITCHI027191.m3*, and *LITCHI027947.m4*, encoding citrate synthase, MDH (oxaloacetate-decarboxylating), mitochondrial pyruvate carrier 2, malate synthase, pyruvate dehydrogenase, and pyruvate dehydrogenase phosphatase, respectively, may significantly contribute to the high acidity of B8 pulps. These genes represent key targets for functional genomics studies toward the molecular breeding of novel litchi varieties with improved quality and market value.

## 4. Materials and Methods

### 4.1. Plant Materials and Sampling

Thirty-year-old litchi trees of two different varieties, Huaizhi (HZ, low-acidity) and Bo8 (B8, high-acidity) located at the National Litchi Germplasm Repository, Guangzhou, China (23°09′14.27″ N; 113°22′15.10″ E), were used in this study. The locality is under a marine subtropical monsoon climate, and the site soil type is red clay soil, suitable for litchi growth. B8 fruit has a short development period, while HZ fruit has a long development period, so the samples were named after the developmental state of the fruits. After pollination and fertilization of a litchi flower, the seed first expands, the seed coat turns brown when the seed approaches its maximum size, and then the aril grows rapidly. Accordingly, the plants were routinely managed, and from 30 days after flowering, a small number of fruits were randomly picked from the tree twice a week to judge the state of development. B8 and HZ fruits were picked at five different developmental stages in 2019. The first stage (S1), representing the young fruit stage was picked when the seed coat turned brown. After that, samples were collected every four days for B8, and weekly for HZ until the fruit was fully ripe. The specific sampling dates are presented in Table S9. According to the pigmentation of the peel, the other stages were named as the expansion stage (S2), green ripening stage (S3), color transformation stage (S4), and red ripening stage (S5) (Figure 1A and Figure S1). When sampling, digital photos of the samples (longitudinal section) were taken. Three biological replicates were set for each variety and thirty fruits were picked at each stage from each tree. The pulps were cut into pieces and mixed equally. A total of 5 × 3 = 15 pulp samples of each variety were collected three times for HPLC, metabolomics, and transcriptomics analyses, respectively. All of the samples were frozen directly in liquid nitrogen and further stored at −80 °C until subsequent analyses. All of the analyses were conducted in triplicate.

### 4.2. HPLC Analysis of Organic Acids

The extraction, detection, and quantification of organic acids was performed following the methods described by Ma et al. with some modification [37]. Briefly, the samples were ground into a fine powder using an IKA A11 grinder cooled with liquid nitrogen. One gram of powder was extracted twice with 4 mL deionized water obtained from a Milli-Q Element. The suspension was centrifuged at 10,000 r/min for 10 min, at 4 °C. Finally, the supernatant from each sample was brought to 10 mL, and 1 mL was taken, then filtered with a 22 μm microporous membrane and used for HPLC (high-performance liquid chromatography) analysis. An HPLC (Agilent 1260 Infinity II) equipped with a 5 μm Titank C18 column (250 mm × 4.6 mm, Guangzhou FLM Scientific Instrument Co., Ltd., Guangzhou, China) was used. The flow rate of the mobile phase (10 mmol/L KH2PO4-H3PO4, pH3.0) was 0.425 mL/min. The column was maintained at 30 °C. Acid was detected with an Agilent G7114A VWD detector at 214 nm. The injection volume was 10 μL. All standards, including malic acid, tartaric acid, citric acid, and VC, were purchased from Yuanye Biotechnology Co., Ltd. (Shanghai, China).

### 4.3. Metabolomics Analysis

The pulp samples were ground for 1.5 min at 30 Hz with a mill (MM400, Retsch, Berlin, Germany). Next, 100 mg of powder of each sample was extracted overnight at 4 °C with 1.0 mL of 70% methanol, followed by 10 min centrifugation at 10,000 g. The supernatants were collected separately and filtered through a 0.22 μm microporous membrane. Then, the extracts were stored in a vial for subsequent UHPLC-MS/MS (ultra-high performance liquid chromatography–mass spectrometry)-based on widely targeted metabolomics analysis. All of the thirty pulp sample extracts were mixed equally to obtain quality control (QC) samples. The LC–MS-gradient-grade solvents methanol, acetonitrile, and acetic acid were purchased from Merck Company (Darmstadt, Hesse, Germany). All other chemicals and standards of metabolites were purchased from BioBioPha (Kunming, Yunnan, China) or Sigma-Aldrich (St. Louis, MO, USA). The standards were dissolved in methanol or dimethyl sulfoxide to obtain standard stock solutions. The standard solutions were stored at −20 °C. Prior to the MS analysis, the standard solutions were diluted with 70% methanol to obtain a gradient of different concentrations.

The data acquisition system mainly included a UHPLC system (Shim-pack UFLC SHIMADZU CBM30A) and a tandem mass spectrometry (MS/MS) system (Applied Biosystems 6500 QTRAP). Metabolites identification and quantification; and multivariate analyses, including hierarchical clustering analysis (HCA), principal component analysis (PCA), K-means analysis, and orthogonal partial least squares discriminant analysis (OPLS-DA), were carried out as per the methods previously reported [38,39]. Briefly, after data quality assessment and standardization via Zscore, the PCA, HCA, and K-means analyses were conducted in R (version 3.5.0) using the statistics function prcomp, pheatmap, and the cluster package (www.r-project.org, accessed on 12 July 2022), respectively. Significant differentially accumulated metabolites (DAMs) were screened out at the thresholds of VIP (variable importance in projection) ≥1 and *t*-test *p* < 0.05. The VIP values were obtained from the OPLS-DA analysis, which was performed on normalized data (log2-transformation) using the R package MetaboAnalystR. Finally, the DAMs were functionally annotated by mapping to the Kyoto Encyclopedia of Genes and Genomes (KEGG) database, followed by significant enrichment analyses [38,39]. Pathways with significantly regulated metabolites were fed into the metabolite sets enrichment analysis (MSEA). Significantly enriched pathways were determined through the hypergeometric test’s *p*-values.

### 4.4. Transcriptomics Sequencing

Total RNA extraction from litchi pulp samples, integrity and quality checking, sequencing on Illumina Hiseq platform, cDNA library construction, and de novo assembly were achieved as per Wang et al. [40] at BGI Genomics Co., Ltd. (Shenzhen, China). The clean reads from each sample were mapped into the litchi reference genome [41] using the TopHat2 software v.2.1.1 with the parameter of no more than one mismatch being accepted in alignment [42]. Gene function annotation was carried out via BLAST against three primary databases: nonredundant protein sequences (NR, http://www.ncbi.nlm.nih.gov, accessed on 30 December 2022, NCBI, National Center for Biotechnology Information), KEGG (http://www.genome.jp/kegg, accessed on 30 December 2022), and the Swiss-Prot protein sequence (http://www.expasy.ch/sprot, accessed on 30 December 2022).

### 4.5. Differentially Expressed Genes (DEGs) Analysis and Functional Annotations

The expression level of each gene was normalized to the number of fragments per kilobase of transcript per million reads using the Cufflinks v.2.0 software [43]. The DEGs were screened out with the DESeq2 software by applying thresholds of |log2Fold Change| ≥ 1 and *p*-value < 0.5 [44]. Functional annotations of DEGs were conducted via GO and KEGG pathway enrichment analyses using the Blast2GO and KOBAS2.0 programs, respectively [45,46].

### 4.6. qRT-PCR Analysis

Fifteen candidate genes were selected randomly for qRT-PCR to confirm the reliability of the RNA-seq data. The analysis was carried out as per Wang et al. [40] on an ABI7500 real-time PCR system (Applied Biosystems). The *Actin* gene served as the internal control for transcripts normalization using the 2^−ΔΔCT^ method [47]. Three biological replicates were applied for each gene. The selected genes and their specific primers are listed in Table S10.

### 4.7. Data Analysis

GraphPad Prism v9.0.0121 (GraphPad 159 Software Inc., La Jolla, CA, USA) was used for statistical analysis and graph construction. Statistical differences were performed by one-way ANOVA at *p* < 0.05. TBtools software v.0.665 was used for gene expression profile heatmapping and qRT-PCR data analysis [48].

## 5. Conclusions

Overall, this study provides deep insights into the molecular mechanisms governing litchi fruits’ acidity variation through comprehensive biochemical, metabolomics, and transcriptomics analyses of developing pulps of low-acid and high-acid litchi varieties. Malic acid is the dominant organic acid in litchi pulps, followed by citric and tartaric acids. During fruit ripening, the content of malic and citric acids was drastically reduced in the low-acid variety. However, in the high-acid variety, the content of citric acid increased, and the malic acid content showed a moderate reduction. The integration of metabolome and transcriptome analyses unveiled key metabolites, genes, and pathways associated with variation in the acidity of litchi fruits. Principally, we found that fumarate and GABA metabolisms are the major driving mechanisms of acidity variation in litchi. The low acidity of litchi pulps is associated with higher induction of GABA and fumarate biosynthesis and accumulation during fruit development, leading to citrate and malate degradation, respectively. Thirty candidate genes were identified for further functional genomics. Of them, fumarase (*LITCHI026501.m2*), glutamate decarboxylase (*LITCHI020148.m5*), and glutamate dehydrogenase (*LITCHI003343.m3*) are the key potential targets for genomics-assisted modulation of litchi fruits’ acidity. Our findings offer valuable resources for litchi fruit quality improvement.

## Figures and Tables

**Table 1:** List of potential candidate genes associated with variation in litchi fruits’ acidity.

Gene_ID	Chr	Gene Length	Log2FC_B8-vs.-HZ					Annotation
			S1	S2	S3	S4	S5	
*LITCHI014080.m1*	Chr2	8049	4.06062	4.07972	3.57043	3.50206	4.81101	Aconitate hydratase
*LITCHI014121.m1*	Chr2	2381	4.21622	5.38847	3.22051	3.18061	3.40981	
*LITCHI005883.m1*	Chr14	10,553	−5.7443	−5.6668	−4.9752	−3.4136	−2.6497	Alcohol dehydrogenase
*LITCHI009351.m2*	Chr7	2030	−4.3612	−8.2589	−4.911	−3.743	−3.1405	
*LITCHI009351.m1*	Chr7	2717	−4.7887	−8.2142	−4.8992	−3.9994	−3.1553	
*LITCHI009351.m4*	Chr7	1613	−5.0403	−5.6369	−4.4061	−4.5642	−3.8755	
*LITCHI014973.m1*	Chr1	7813	−2.3199	−1.8379	−3.1671	−2.699	−2.8939	ATP citrate (pro-S)-lyase
*LITCHI015351.m4*	Chr1	2941	3.24125	2.79842	3.06652	2.04997	−1.1406	
*LITCHI004325.m6*	Chr14	5042	2.57725	−4.5858	NA	−1.2316	−0.1214	Citrate synthase
*LITCHI020148.m5*	Chr12	7582	0.84976	2.50526	1.33812	1.30628	3.19904	Glutamate decarboxylase
*LITCHI003343.m3*	Chr6	2915	0.28903	2.36364	4.60759	4.35457	3.36485	Glutamate dehydrogenase (NAD(P)+)
*LITCHI014417.m1*	Chr1	766	−7.4384	−7.6799	−7.6302	−5.9068	−4.2463	Glutamate receptor, ionotropic, plant
*LITCHI018688.m1*	Chr15	10,459	7.74389	6.88227	3.17553	6.89497	6.98392	Glutamine synthetase
*LITCHI017210.m2*	Chr1	4944	−3.8161	−4.6483	−4.8736	−5.3081	−3.0973	Malate dehydrogenase (oxaloacetate-decarboxylating) (NADP+)
*LITCHI017210.m4*	Chr1	3658	5.80302	3.57723	3.80498	−1.4727	−0.0223	
*LITCHI017210.m3*	Chr1	3658	5.80302	3.57723	3.80498	−1.4727	−0.0223	
*LITCHI006460.m2*	Chr11	3827	1.20331	2.45027	0.9708	2.73029	3.01809	Malate dehydrogenase
*LITCHI021353.m1*	Chr12	1611	−5.6461	−7.5779	−6.5181	−3.9782	−2.512	Malate synthase
*LITCHI014881.m2*	Chr1	685	−1.8423	−3.6235	−2.9322	−3.2504	−2.7075	Mitochondrial pyruvate carrier 2
*LITCHI012899.m9*	Chr2	2105	2.28868	NA	4.47104	0.66693	−5.3621	Phosphoenolpyruvate carboxylase
*LITCHI005456.m2*	Chr14	15,180	−1.1193	−1.4388	−0.656	0.06627	7.75064	
*LITCHI027191.m3*	Chr3	7665	−5.1948	−6.1722	−7.9115	−8.4677	−8.1616	Pyruvate dehydrogenase
*LITCHI027947.m4*	Chr3	3598	−2.4055	−1.1367	−0.0642	−3.5808	−2.4764	Pyruvate dehydrogenase phosphatase
*LITCHI014910.m2*	Chr1	4544	0.58152	−2.1858	−4.5882	−7.1461	−4.1166	Pyruvate kinase
*LITCHI006026.m1*	Chr14	7987	5.73614	5.63149	5.63661	5.5364	5.40993	
*LITCHI010636.m1*	Chr8	975	2.35935	2.91427	−2.8211	−1.5014	1.12691	Solute carrier family 25 (mitochondrial oxoglutarate transporter)
*LITCHI007011.m3*	Chr11	2142	0.83409	3.09493	0.71148	−1.2857	2.30272	
*LITCHI013926.m3*	Chr2	3341	NA	NA	0.4415	3.81511	2.8162	2-oxoglutarate dehydrogenase
*BGI_novel_G000750*	Chr4	6972	4.11965	1.87247	1.78248	2.097	0.82182	Succinyl-CoA synthetase
*LITCHI026501.m2*	Chr3	5631	3.79998	7.99249	7.43426	7.79962	7.75682	Fumarate hydratase

## Data Availability

The dataset is available at the SRA (https://www.ncbi.nlm.nih.gov/sra/PRJNA880983, accessed on 30 December 2022) under the accession number of PRJNA880983.

## Supplementary Materials

The supporting information can be downloaded at: https://www.mdpi.com/article/10.3390/ijms24031871/s1.
