# Supplementary material for: Combined Metabolome and Transcriptome Analyses Unveil the Molecular Mechanisms of Fruit Acidity Variation in Litchi (Litchi chinensis Sonn.)
Source: Int J Mol Sci. 2023 Jan 18;24(3):1871. doi: 10.3390/ijms24031871 (PMC9916176; doi:10.3390/ijms24031871)
Supplement: Supplementary file 1 [file ijms-24-01871-s001.zip › Supplementary Figures.pdf]

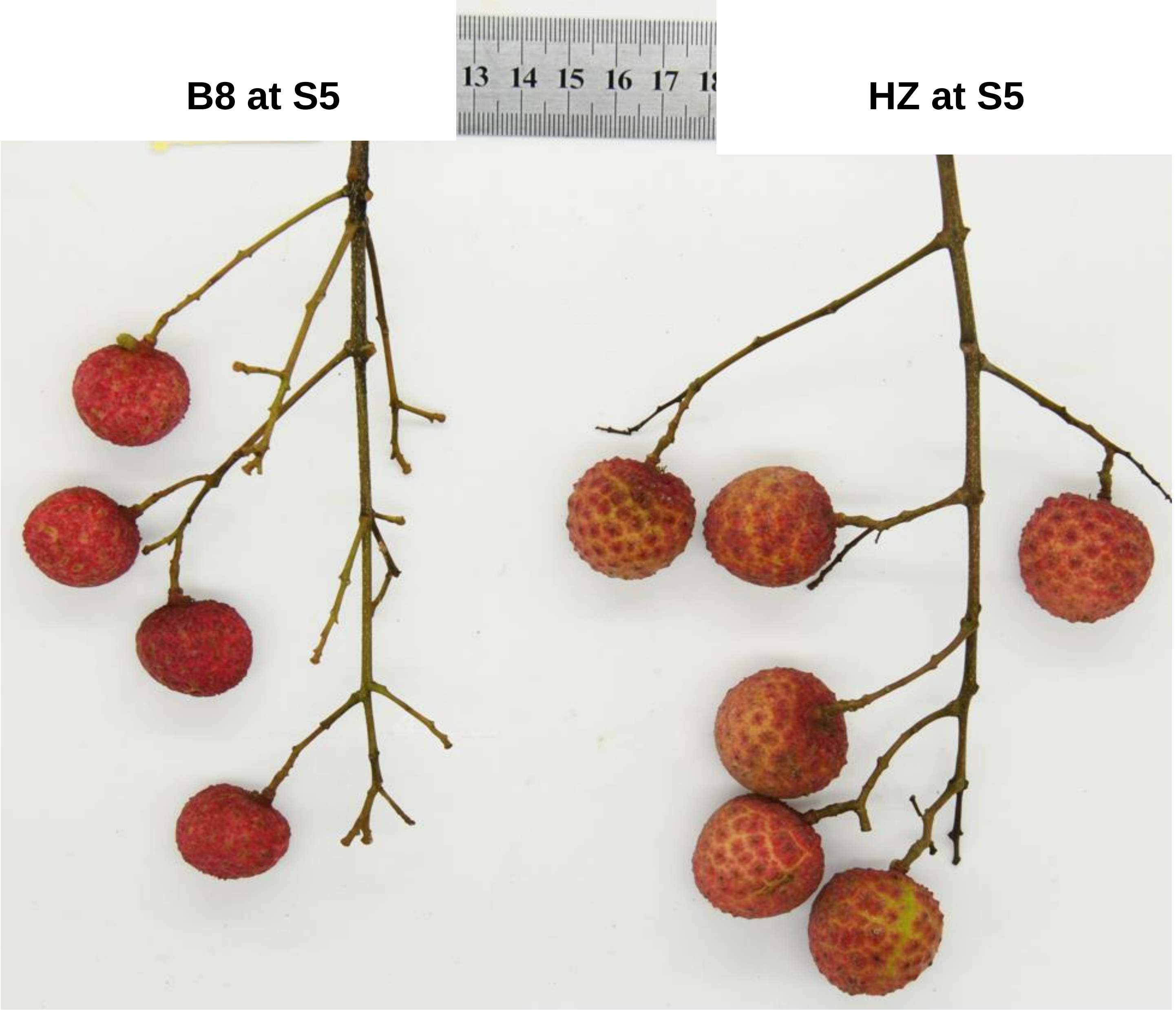

Figure S1. Representative images of HZ and B8 fruits at S5.

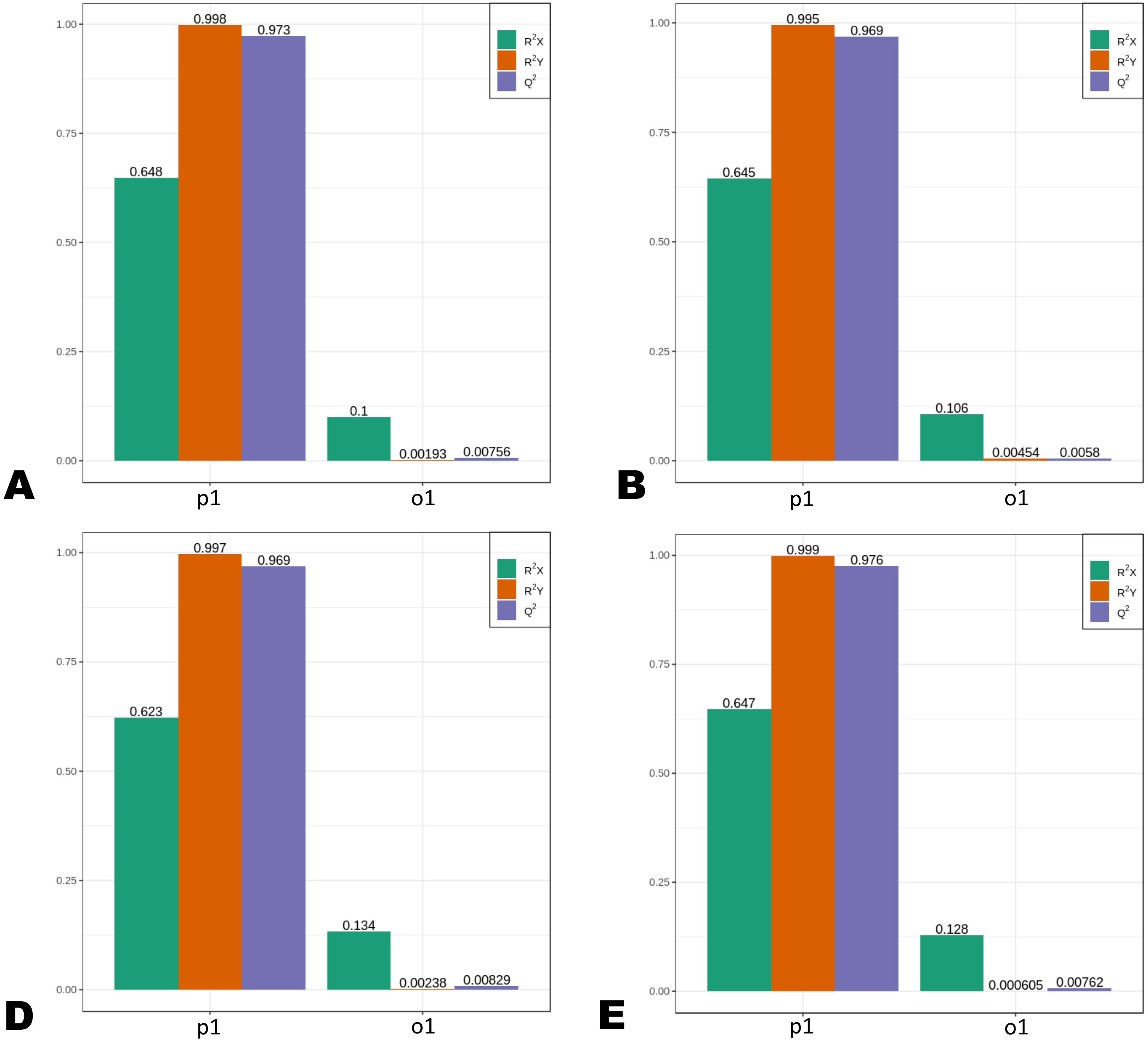

Figure S2. OPLS-DA analysis results of pairwise comparison between HZ and B8 at S1, S2, S3, S4, and S5, respectively.

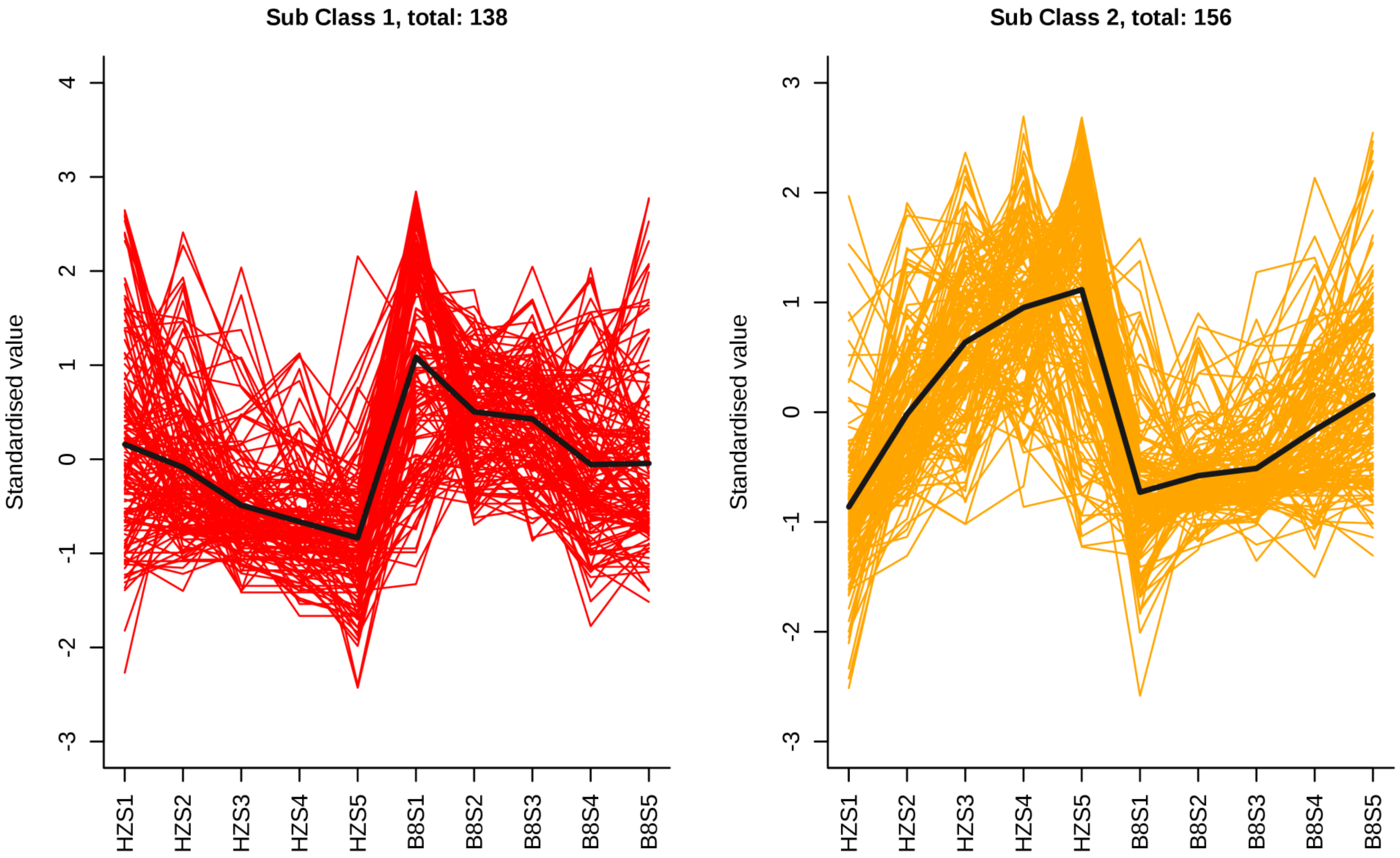

Figure S3. K-mean analysis result of metabolites interlinked with organic acid metabolism.

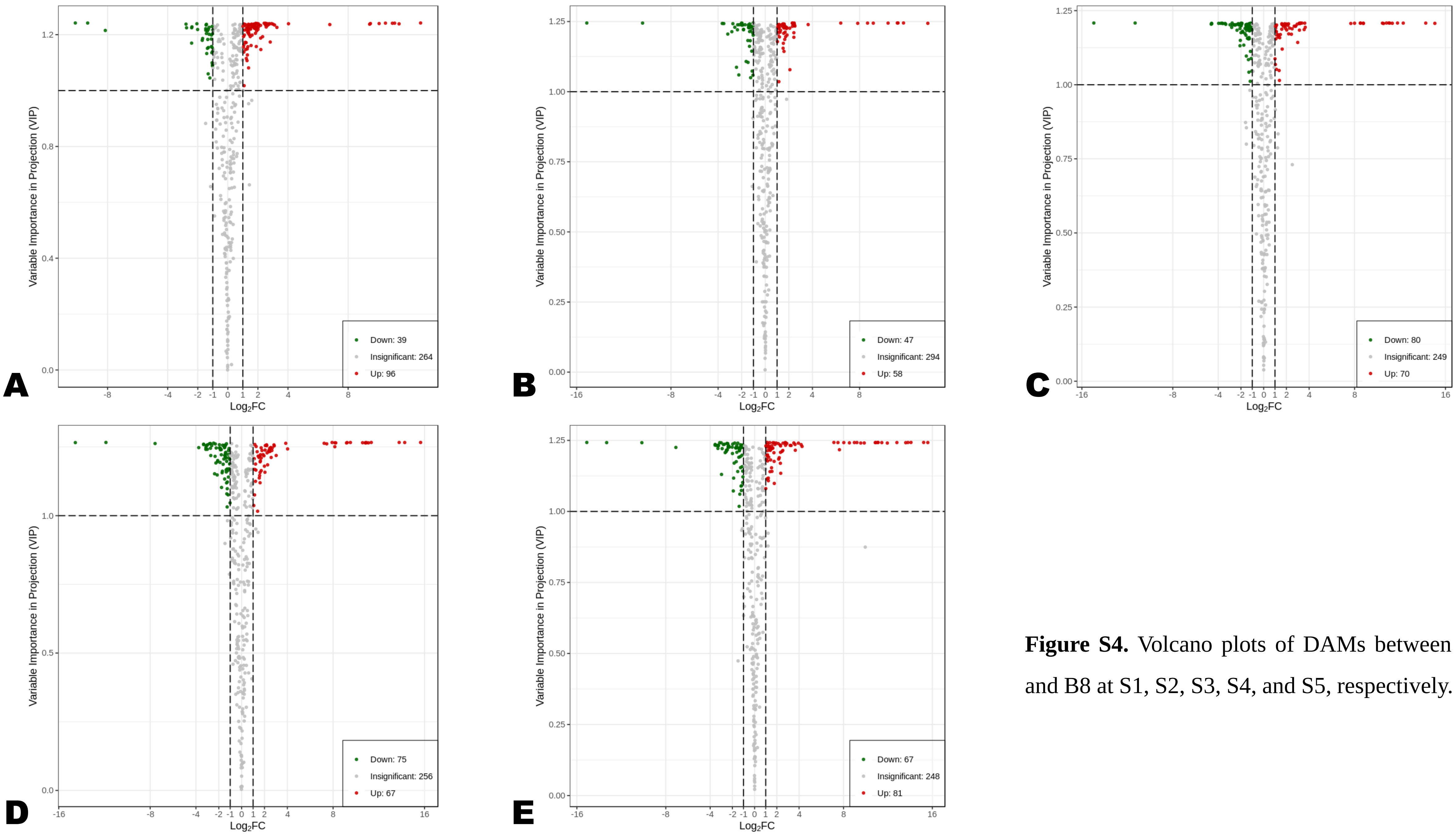

Figure S4. Volcano plots of DAMs between HZ and B8 at S1, S2, S3, S4, and S5, respectively.

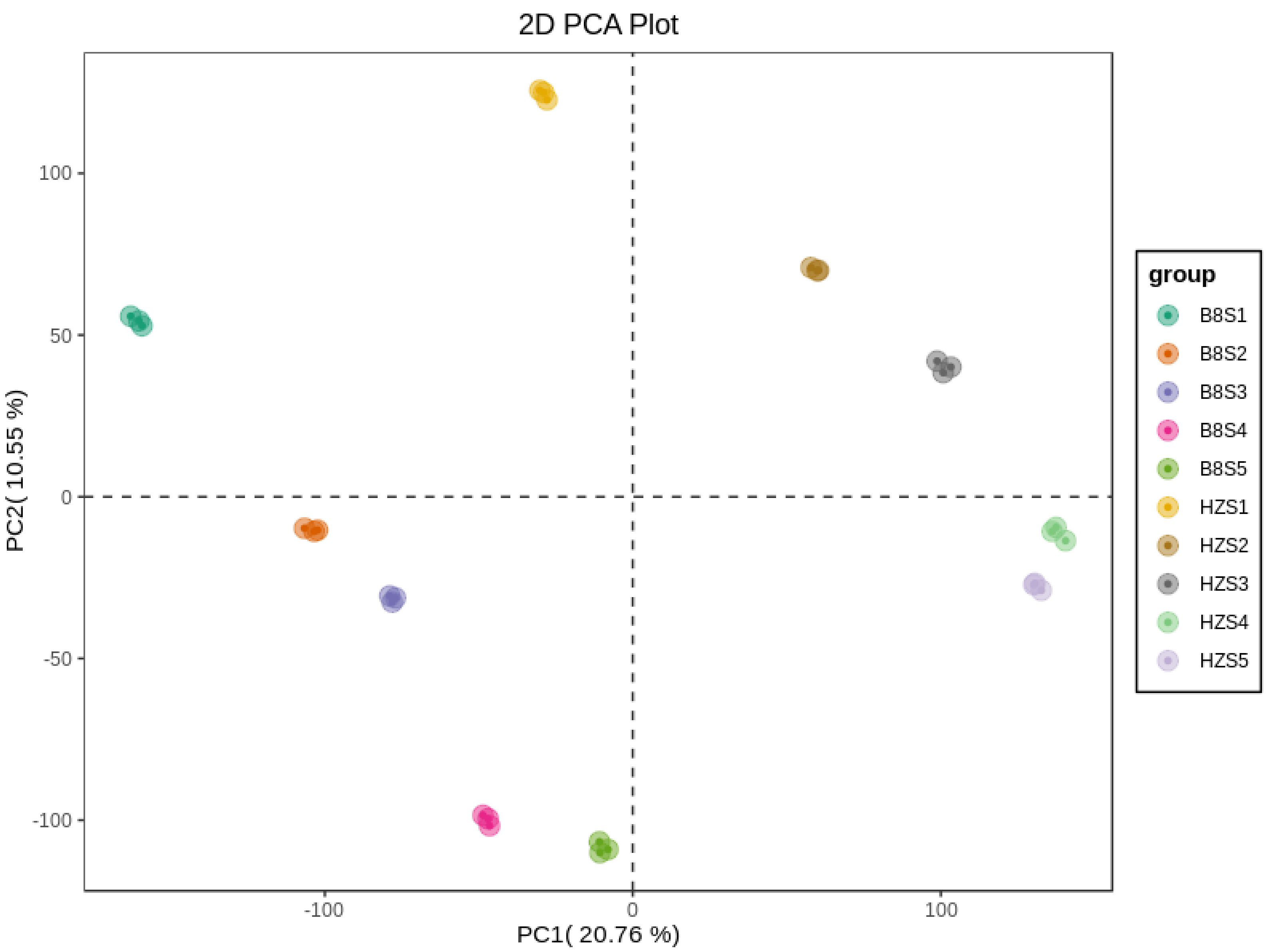

Figure S5. Principal component analysis of transcriptome of HZ and B8 pulps at different development stages.

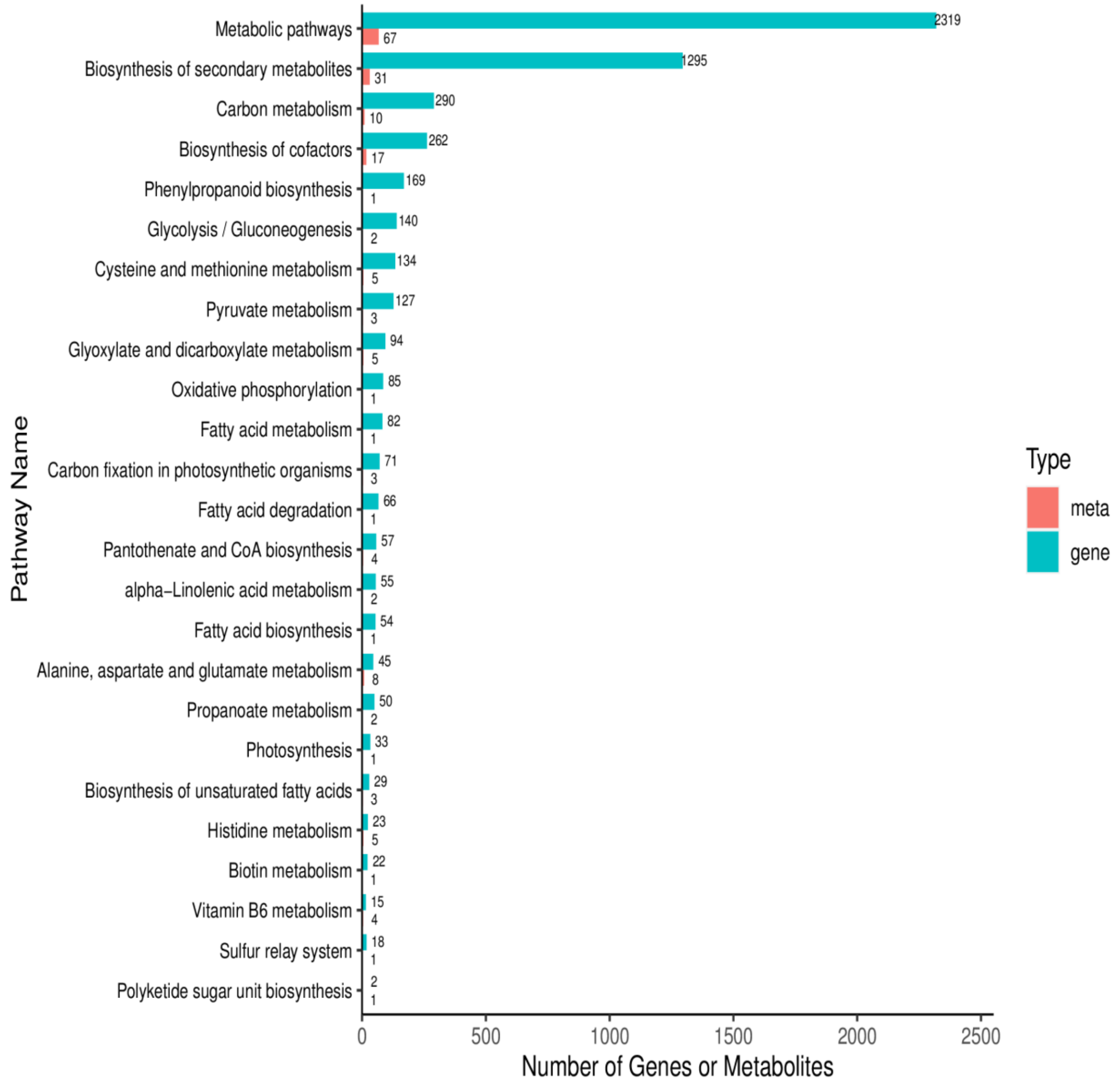

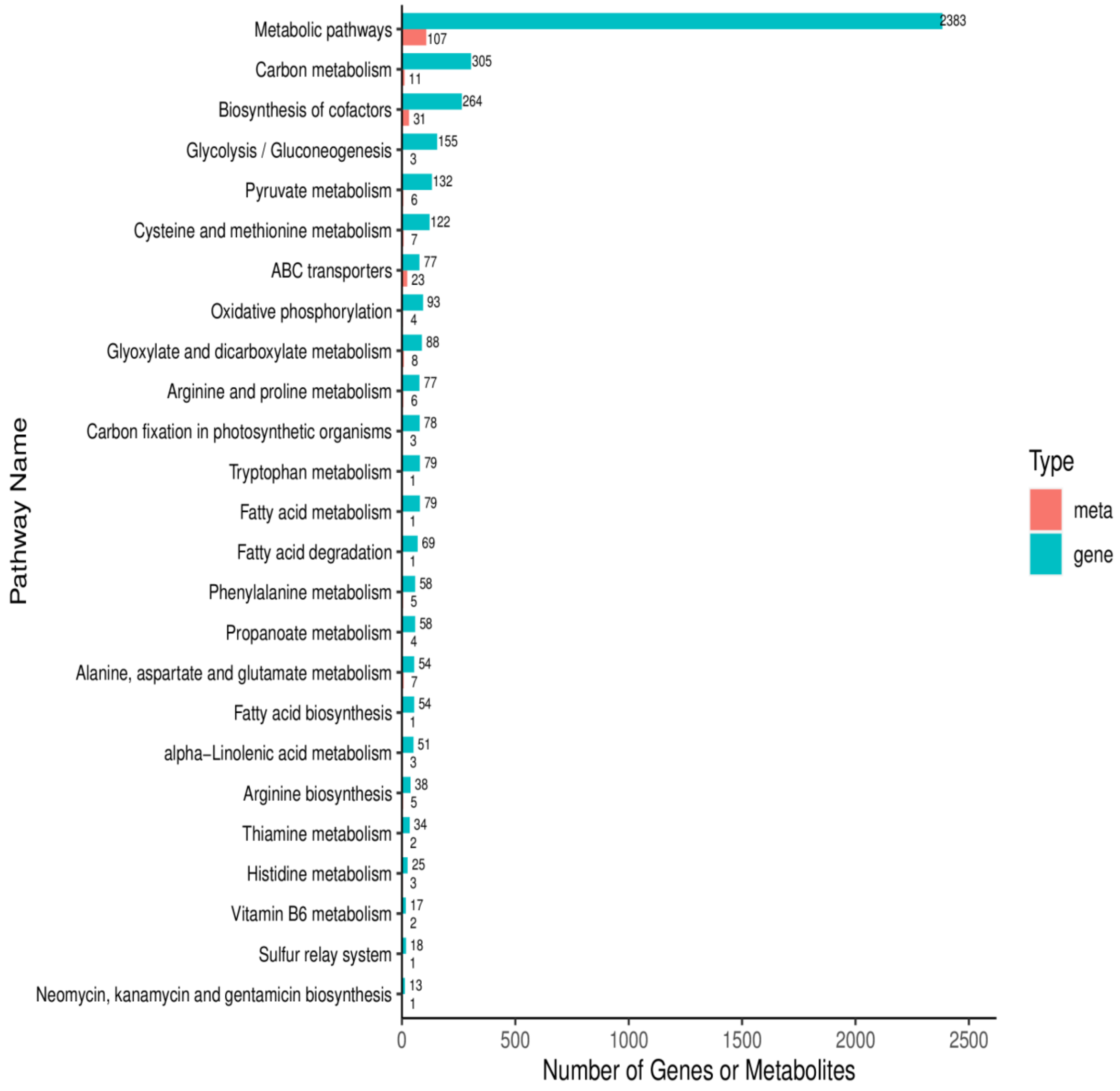

Figure S6. Differentially expressed genes (DEGs) between HZ and B8 during fruit repining. (A) Summary of the number of DEGs during fruit development in B8 and HZ, and between the two varieties. (B) Venn diagram of DEGs between HZ and B8 at the five fruit developmental stages. (C) and (D) KEGG annotation results of the DEGs between B8S1 and B8S5; and between HZS1 and HZS5, respectively.

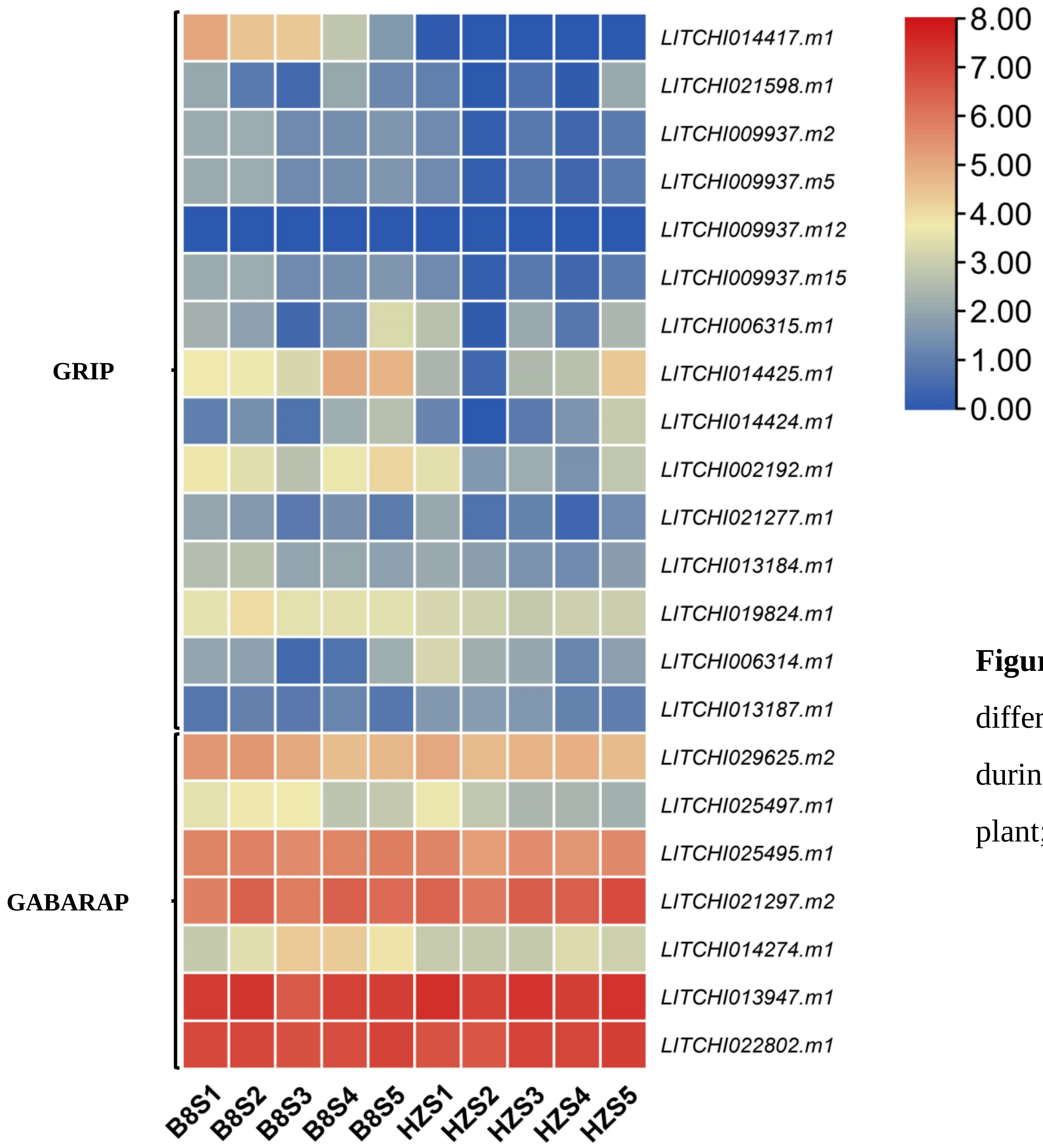

Figure S7. Heatmap of log2FC values of the expression of key differentially expressed organic acid metabolism genes in HZ and B8 during fruit development. GRIP, Glutamate receptor, ionotropic, plant; GABARAP, GABA receptors.

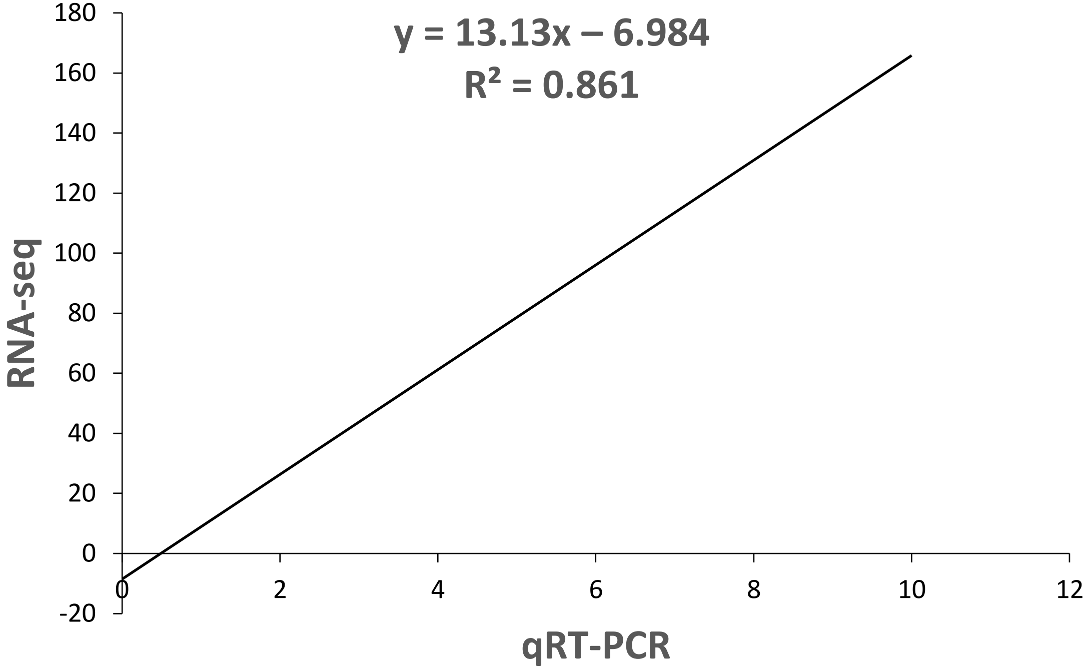

Figure S8. qRT-PCR validation of fifteen selected candidate genes.
